# Association of hospital-based substance use supports on emergency department revisits: a retrospective cohort study in Sudbury, Canada from 2018 to 2022

**DOI:** 10.1186/s12954-024-00985-0

**Published:** 2024-03-28

**Authors:** Mark Tatangelo, Russell Landry, Denis Beaulieu, Catherine Watson, Shannon Knowlan, Alex Anawati, Adele Bodson, Natalie Aubin, David C. Marsh, Tara Leary, Kristen A. Morin

**Affiliations:** 1 Health Science North, Sudbury, ON Canada; 2 ICES North, Sudbury, ON Canada; 3 Dr. Gilles Arcand Centre for Health Equity, Sudbury, ON Canada; 4 Northern Ontario School of Medicine University, Sudbury, ON Canada; 5 Laurentian University, Sudbury, ON Canada

**Keywords:** Harm-reduction, Emergency department revisits, Substance use disorders, Addiction medicine, Addiction consult teams, Observational data, Administrative data, Cohort study

## Abstract

**Background:**

This study compares emergency department (ED) revisits for patients receiving hospital-based substance-use support compared to those who did not receive specialized addiction services at Health Sciences North in Sudbury, Ontario, Canada.

**Methods:**

The study is a retrospective observational study using administrative data from all patients presenting with substance use disorder (SUD) at Health Sciences North from January 1, 2018, and August 31, 2022 with ICD-10 codes from the Discharge Abstract Database (DAD) and the National Ambulatory Care Database (NACRS). There were two interventions under study: addiction medicine consult services (AMCS group), and specialized addiction medicine unit (AMU group). The AMCS is a consult service offered for patients in the ED and those who are admitted to the hospital. The AMU is a specialized inpatient medical unit designed to offer addiction support to stabilize patients that operates under a harm-reduction philosophy. The primary outcome was all cause ED revisit within 30 days of the index ED or hospital visit. The secondary outcome was all observed ED revisits in the study period. Kaplan–Meier curves were used to measure the proportion of 30-day revisits by exposure group. Odds ratios and Hazard Ratios were calculated using logistic regression models with random effects and Cox-proportional hazard model respectively.

**Results:**

A total of 5,367 patients with 10,871 ED index visits, and 2,127 revisits between 2018 and 2022 are included in the study. 45% (2,340/5,367) of patient were not admitted to hospital. 30-day revisits were less likely among the intervention group: Addiction Medicine Consult Services (AMCS) in the ED significantly reduced the odds of revisits (OR 0.53, 95% CI 0.39–0.71, *p* < 0.01) and first revisits (OR 0.42, 95% CI 0.33–0.53, *p* < 0.01). The AMU group was associated with lower revisits odds (OR 0.80, 95% CI 0.66–0.98, *p* = 0.03). For every additional year of age, the odds of revisits slightly decreased (OR 0.99, 95% CI 0.98–1.00, *p* = 0.01) and males were found to have an increased risk compared to females (OR 1.50, 95% CI 1.35–1.67, *p* < 0.01).

**Interpretation:**

We observe statistically significant differences in ED revisits for patients receiving hospital-based substance-use support at Health Sciences North. Hospital-based substance-use supports could be applied to other hospitals to reduce 30-day revisits.

**Supplementary Information:**

The online version contains supplementary material available at 10.1186/s12954-024-00985-0.

## Introduction

Canadian data shows the age-adjusted emergency department (ED) visit rate due to opioid poisoning in the province of Ontario rose by 47% from 2012 to 2016. The costs and intensity of visits to ED and hospital for substance use disorder (SUD) are notable. This has led to an increase in substance use-related ED visits and a rising prevalence of substance use [1–3]. For example, hospitalizations attributable to alcohol-related issues cost $8,100, compared to an average hospitalization cost of $5,800 [4].

Policymakers and hospitals in Ontario are designing new interventions to reduce high rates ED and hospitalizations among SUD populations because patients with SUD may benefit from targeted hospital-based substance use support [5–8]. Since revisits to ED are a proxy to measure effective treatment for SUD, and a direct indicator for increased health system use [9], interventions that may modify the occurrence of revisit events could improve patient care and reduce health resource costs. Research studies indicate that flexible, harm-reduction focused addiction-specific services in acute care settings have the potential to enhance the quality of care and improve outcomes for patients during hospitalizations [10–12]. However, considerable heterogeneity among populations studied, the types of interventions implemented, and the outcomes evaluated limits conclusions or established recommendations.

Health Sciences North (HSN), an academic health sciences centre in Sudbury, Ontario, Canada has implemented harm-reduction focused hospital-based SUD supports. The primary interventions are the Addiction Medicine Consult Services (AMCS), which provide addiction-specific consultations and interventions to all patients in the ED and admitted to the hospital, and the Addiction Medicine Unit (AMU), a specialized inpatient medical unit designed to offer addiction support to stabilize patients. The main objective of the AMU is to stabilize patients and provide targeted services such as managing withdrawal, addressing cravings, and offering opioid agonist treatment, while meeting people where they are in their substance use journey.

This study aims to measure the association between patients receiving hospital-based substance use support upon ED or hospital admission to HSN, revisits within 30 days and any revisits from 2018 to 2022 compared to the standard of care.

## Methods

### Design and setting

This is a retrospective cohort study of patients who had an index revisit to the ED at HSN in Sudbury, Ontario, Canada with substance use as the primary or secondary reason for visit (F10-19 within ICD-10-CA Chapter 5) [13]. All data were de-identified with consent waiver, in compliance with local ethics and privacy laws (PIPEDA, Personal Health Information Protection Act, TCPS2 5.4D), reviewed by the Health Sciences North Research Ethics Board. Research was conducted in accordance with the Tri-Council Policy Statement Ethical Conduct for Research Involving Humans in Canada.

HSN is an acute care hospital located in Sudbury, Canada, which is considered a small urban setting in Northern Ontario serving approximately 570,000 people across Northeastern Ontario [14]. Data is from administrative sources which contain no missing data.

### Data sources and study population

**Fig. 1:**
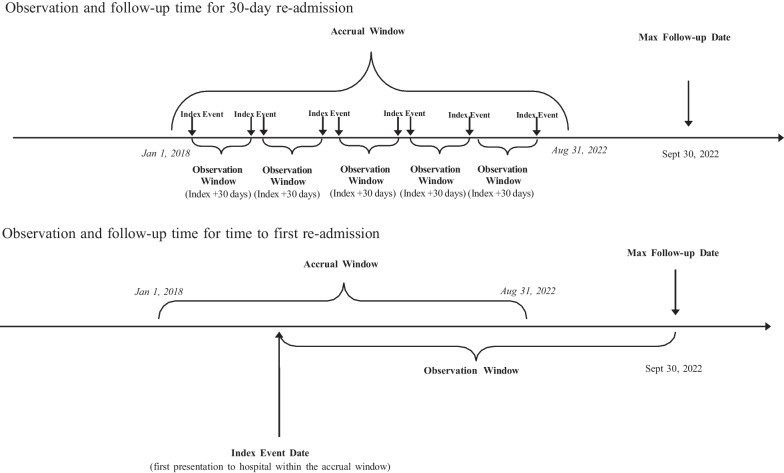
Study diagram for 30-day re-admission and first readmission

Study participants were identified from medical records at HSN between January 1, 2018, and August 31, 2022, with patient outcome accrual ending on September 30th, 2022 (Fig. 1). The Discharge Abstract Database (DAD) [15] contains detailed information on all hospital admission and discharges, and the National Ambulatory Care Reporting System database (NACRS) [16] contains information on hospital ED visits and discharges including ICD-10 [17] diagnosis codes were used as source data for the study.

### Interventions

Two in-hospital intervention groups were studied: (1) the AMCS group: which provides substance use support to all patients both hospitalized and in ED; (2) the AMU group: an inpatient medical unit designed to offer addiction support to stabilize patients. For both interventions, patients were referred to the intervention by the admitting physician based on clinical criteria of indication for SUD. To be admitted to AMU, patients are required to have an acute medical or psychiatric diagnosis and require ongoing care with concurrent active addiction concerns, or acute withdrawal requiring medical monitoring outside the ICU.

Two standard-of-care groups were used as comparators: (1) The ED visit group (reference group): patients who presented to the ED and were discharged directly from the ED without receiving addiction support (2) the admit/no service group: patients who were admitted to an inpatient unit in the hospital but did not receive specialized addiction services. All groups were compared to the reference group (ED no services group).

### Index event

Index events are defined as the discharge date from hospital or ED for a primary or secondary diagnosis of SUD with DAD or NACRS discharge codes of F10-19 within ICD-10-CA Chapter 5) [13].

*Primary outcome* (Revisit to ED within 30-days of an index event).

The study outcomes are defined a priori as visit to ED within 30-days of an index event [8]. 30-day ED revisits are defined as all cause visit to ED within 30-days of the index visit. The 30-day window starts when the index visit discharge date occurred. If a revisit does not occur within 30-days of the index date, the 30-day window is re-started upon the presentation which becomes the new index date (Fig. 1).

*Secondary outcome* (First revisit to ED after first index event).

The secondary outcome was first all cause revisit to ED after the first index event occurring within the study period. If no revisit event occurred within the study period, the patients were considered left censored.

### Covariates

Covariates for the study were collected at the time of admission to ED visit and are considered as baseline covariates. Age, biological sex, homelessness, and visits to the ED or hospital for mental health, and primary or tertiary occurrence of alcohol or opioid ICD-10 codes [17]. Two criteria were used to identify homelessness for this study: (1) patients were flagged by identifying an ICD-10 code Z59; (2) trained abstractors examine the EMR for physician notes of homelessness, and descriptors of homelessness (no fixed address). Mental health diagnoses were determined using all ICD-10 F codes, excluding F1, which indicates substance use. Opioids and alcohol were determined using the occurrence of ICD-10 codes: Alcohol F10 and Opioid F11 as primary or tertiary diagnosis.

Comorbidities events were measured from ICD-10-CM codes and grouped into 31 clinically meaningful groups using the Clinical Classifications Software Refined (CCSR) for ICD-10-CM; a diagnostic categorization tool developed by the Agency for Healthcare Research and Quality (AHRQ).^1^

### Statistical analysis

Descriptive analysis to summarize the baseline characteristics of our study population for both continuous and categorical variables [18]. For continuous variables, we reported the mean and standard deviation. Categorical variables were summarized using frequencies and percentages. Logistic regression models were used to determine the association of the main interventions and covariates with the outcomes. For the 30-day windows, random effects adjusting for a within-patient clustering were included [19]. In addition to logistic regression models, Cox proportional hazards models [20, 21] were used to investigate the main interventions and covariate factors associated with time to revisits within 30-days and time to first revisit. For revisit within 30 days, random effects for within-patient variance were fit. All statistical tests were at the *p* = 0.05 and 95% confidence threshold for statistical significance. Kaplan–Meier curves were fit to measure the raw revisit probabilities within 30-days of index admission, and the time to first revisit within the first year after index admission. Both Kaplan–Meier curves were tested for differences with the Mantel–Haenszel test.^2^ All statistical analyses were computed in R version 4.2.2 [22].

## Results

**Fig. 2:**
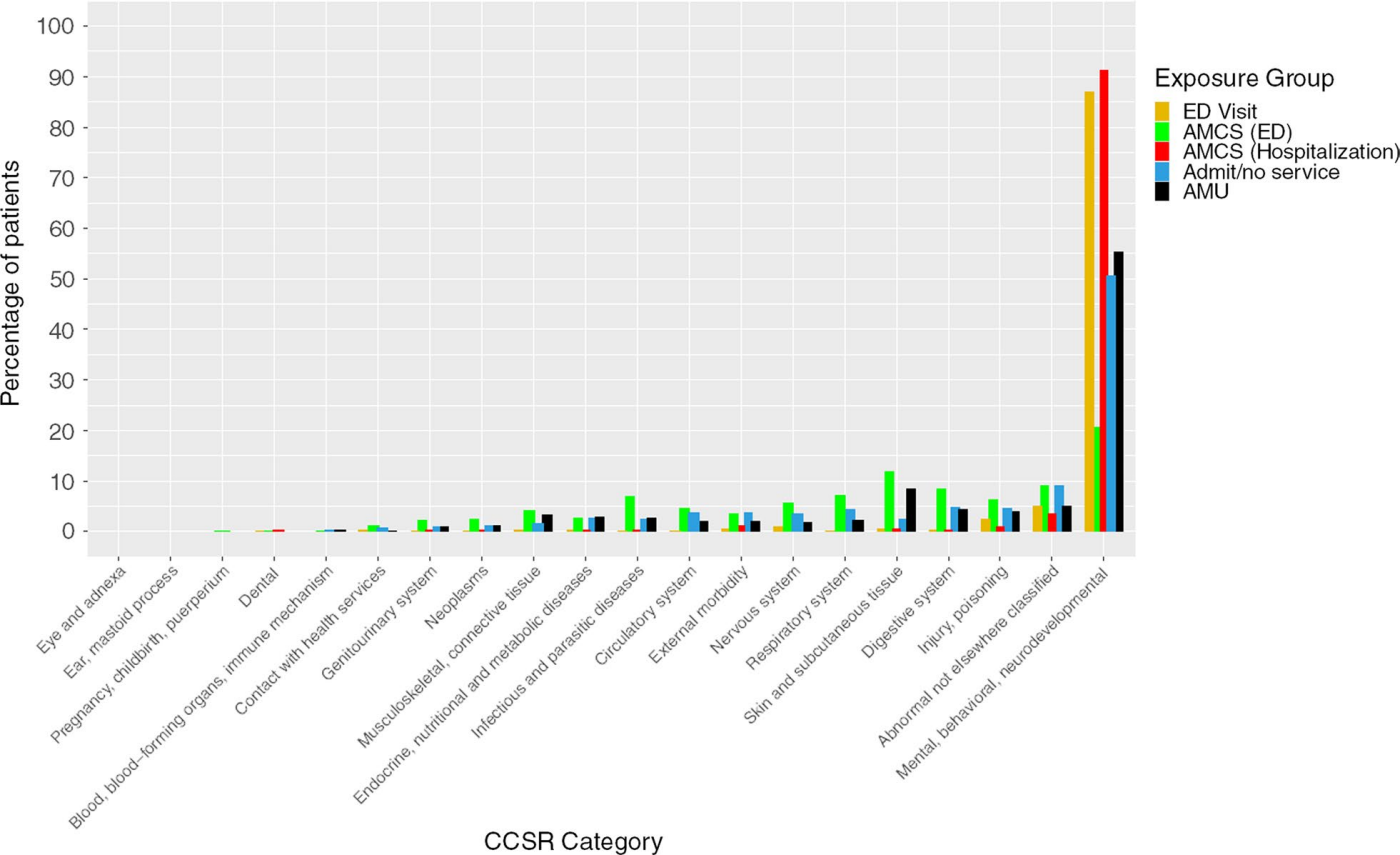
Clinical Classifications Software Refined (CCSR) Comorbidities per patient at Hospital Grouped by Exposure

A total of 5,267 patients with 10,871 index events and 2,127 outcome events were observed for a 19.6% (2,127/10,871) crude 30-day ED revisit event rate. The mean follow-up time was 39.56 days for all index events (Table 1A), 826.67 for first index events (Table 1B) and 9.21 days for outcome events (Table 1C). In the ED standard of care group, 2,340 patients, accrued 4,929 index events and 1,303 outcome events. Meaning that 45% (2,340/5,367) of patient were not admitted to hospital. The AMCS ED intervention group contains 4,313 patients, with 303 index events and 97 outcome events. The AMCS group has 314 patients, 716 index events, and 82 outcome events. The standard of care admitted/no services group had 2,497 patients, 4,313 index events, and 507 outcome events. The AMU intervention group 130 patients incurred 610 index events and 138 outcome events. The primary reason for the first index visit at baseline for this cohort was mental health or substance-related and this was true for over 50% of the cohort (Fig. 2 and Additional file 1).

**Table 1:** Patient characteristics

Variable	Overall	ED visit (reference)	AMCS (ED)	AMCS (hospitalization)	Admit no service	AMU	*p*
*Panel A: for all index events*							
n	10,871	4,929	303	716	4,313	610	
Follow-Up Time (mean (SD))	39.56 (16.01)	36.78 (14.62)	35.31 (11.61)	46.20 (15.13)	41.81 (17.65)	40.46 (12.01)	< 0.01
Age (mean (SD))	7091 (65.2)	3227 (65.5)	180 (59.4)	472 (65.9)	2806 (65.1)	406 (66.6)	0.254
Sex = Male (%)	1873 (17.2)	825 (16.7)	51 (16.8)	123 (17.2)	739 (17.1)	135 (22.1)	0.025
Homeless (%)	2057 (18.9)	499 (10.1)	194 (64.0)	13 (1.8)	1333 (30.9)	18 (3.0)	< 0.01
Mental Health Diagnosis (ED) (%)	2057 (18.9)	499 (10.1)	194 (64.0)	13 (1.8)	1333 (30.9)	18 (3.0)	< 0.01
Mental Health Diagnosis (Inpatient) (%)	1164 (10.7)	8 (0.2)	0 (0.0)	173 (24.2)	766 (17.8)	217 (35.6)	< 0.01
Opioid-related Diagnosis (ED) (mean (SD))	1.64 (1.08)	1.66 (1.11)	1.74 (1.36)	1.80 (1.20)	1.60 (1.01)	1.52 (0.96)	0.409
Opioid-related Diagnosis (Inpatient) (mean (SD))	3.77 (3.22)	3.83 (3.26)	4.39 (3.49)	3.80 (3.04)	3.67 (3.05)	3.58 (3.75)	0.764
Alcohol-related Diagnosis (ED) (mean (SD))	1.52 (1.08)	1.52 (1.14)	1.65 (1.40)	1.66 (1.16)	1.47 (0.98)	1.55 (1.08)	0.064
Alcohol-related Diagnosis (Inpatient) (mean (SD))	3.51 (2.83)	3.53 (2.87)	3.33 (2.78)	3.82 (3.11)	3.47 (2.72)	3.32 (2.98)	0.615
*Panel B: for each patient (first index event)*							
n	5,367	2,340	86	314	2,497	130	
Follow-Up Time (mean (SD))	826.67 (509.69)	834.26 (533.10)	392.15 (329.67)	570.60 (287.56)	895.86 (490.71)	267.11 (164.46)	< 0.01
Age (mean (SD))	39.70 (17.54)	34.59 (15.05)	34.42 (12.36)	48.24 (15.83)	43.48 (18.77)	41.83 (14.42)	< 0.01
Sex = Male (%)	3359 (62.6)	1450 (62.0)	47 (54.7)	200 (63.7)	1580 (63.3)	82 (63.1)	0.49
Homeless (%)	890 (16.6)	372 (15.9)	11 (12.8)	52 (16.6)	425 (17.0)	30 (23.1)	0.19
Mental Health Diagnosis (ED) (%)	1052 (19.6)	275 (11.8)	61 (70.9)	4 (1.3)	705 (28.2)	7 (5.4)	< 0.01
Mental Health Diagnosis (Inpatient) (%)	594 (11.1)	4 (0.2)	0 (0.0)	77 (24.5)	472 (18.9)	41 (31.5)	< 0.01
Opioid-related Diagnosis (ED) (mean (SD))	1.61 (1.06)	1.59 (1.13)	2.00 (1.88)	1.97 (1.15)	1.56 (0.92)	1.39 (0.92)	0.12
Opioid-related Diagnosis (Inpatient) (mean (SD))	3.94 (3.23)	4.16 (3.21)	4.00 (2.24)	3.47 (3.44)	3.78 (3.21)	4.00 (3.80)	0.72
Alcohol-related Diagnosis (ED) (mean (SD))	1.50 (1.06)	1.51 (1.14)	1.83 (1.83)	1.58 (0.89)	1.46 (0.96)	1.66 (1.13)	0.27
Alcohol-related Diagnosis (Inpatient) (mean (SD))	3.54 (2.85)	3.54 (2.86)	2.75 (2.34)	3.95 (3.58)	3.52 (2.76)	3.17 (2.59)	0.58
*Panel C: for outcome events*							
n	2,127	1,303	97	82	507	138	
Follow-Up Time (mean (SD))	9.21 (8.61)	7.98 (8.47)	11.10 (8.71)	10.01 (7.60)	11.49 (8.64)	10.69 (8.21)	< 0.01
Age (mean (SD))	39.38 (13.54)	40.14 (13.94)	43.10 (13.17)	35.80 (10.29)	37.03 (13.24)	40.40 (11.05)	< 0.01
Sex = Male (%)	1537 (72.3)	954 (73.2)	72 (74.2)	49 (59.8)	366 (72.2)	96 (69.6)	0.10
Homeless (%)	352 (16.5)	209 (16.0)	18 (18.6)	11 (13.4)	85 (16.8)	29 (21.0)	0.53
Mental Health Diagnosis (ED) (%)	318 (15.0)	82 (6.3)	2 (2.1)	46 (56.1)	184 (36.3)	4 (2.9)	< 0.01
Mental Health Diagnosis (Inpatient) (%)	143 (6.7)	1 (0.1)	24 (24.7)	0 (0.0)	69 (13.6)	49 (35.5)	< 0.01
Opioid-related Diagnosis (ED) (mean (SD))	1.71 (1.16)	1.82 (1.24)	1.88 (1.13)	1.12 (0.35)	1.51 (0.92)	1.79 (1.40)	0.28
Opioid-related Diagnosis (Inpatient) (mean (SD))	3.67 (3.23)	3.55 (2.81)	5.00 (3.44)	5.12 (4.52)	3.34 (2.74)	3.50 (5.35)	0.38
Alcohol-related Diagnosis (ED) (mean (SD))	1.50 (1.10)	1.52 (1.20)	1.65 (0.92)	1.38 (1.02)	1.40 (0.88)	1.60 (1.05)	0.53
Alcohol-related Diagnosis (Inpatient) (mean (SD))	3.56 (2.96)	3.50 (2.92)	5.10 (3.05)	3.67 (3.72)	3.32 (2.26)	3.58 (4.14)	0.17

*SD* standard deviation, *ED* emergency department, *AMCS (hospitalization)* Addiction medicine consult service during hospital admission, *AMU* Addiction medicine unit

Statistically significant differences between the intervention groups observed in 30-day windows for all index events included follow-up time (*p* < 0.01), homelessness (*p* < 0.025) and diagnosis for mental health in ED and inpatient (*p* < 0.01) (Table 1 Panel A).

Time to first revisit and outcome events only showed statistically significant differences between the groups on age (*p* < 0.01), and follow-up time (*p* < 0.01), and mental health ED visits and hospital admissions (*p* < 0.01) (Table 1 Panel B, and Panel C).

### Logistic regression results

**Fig. 3:**
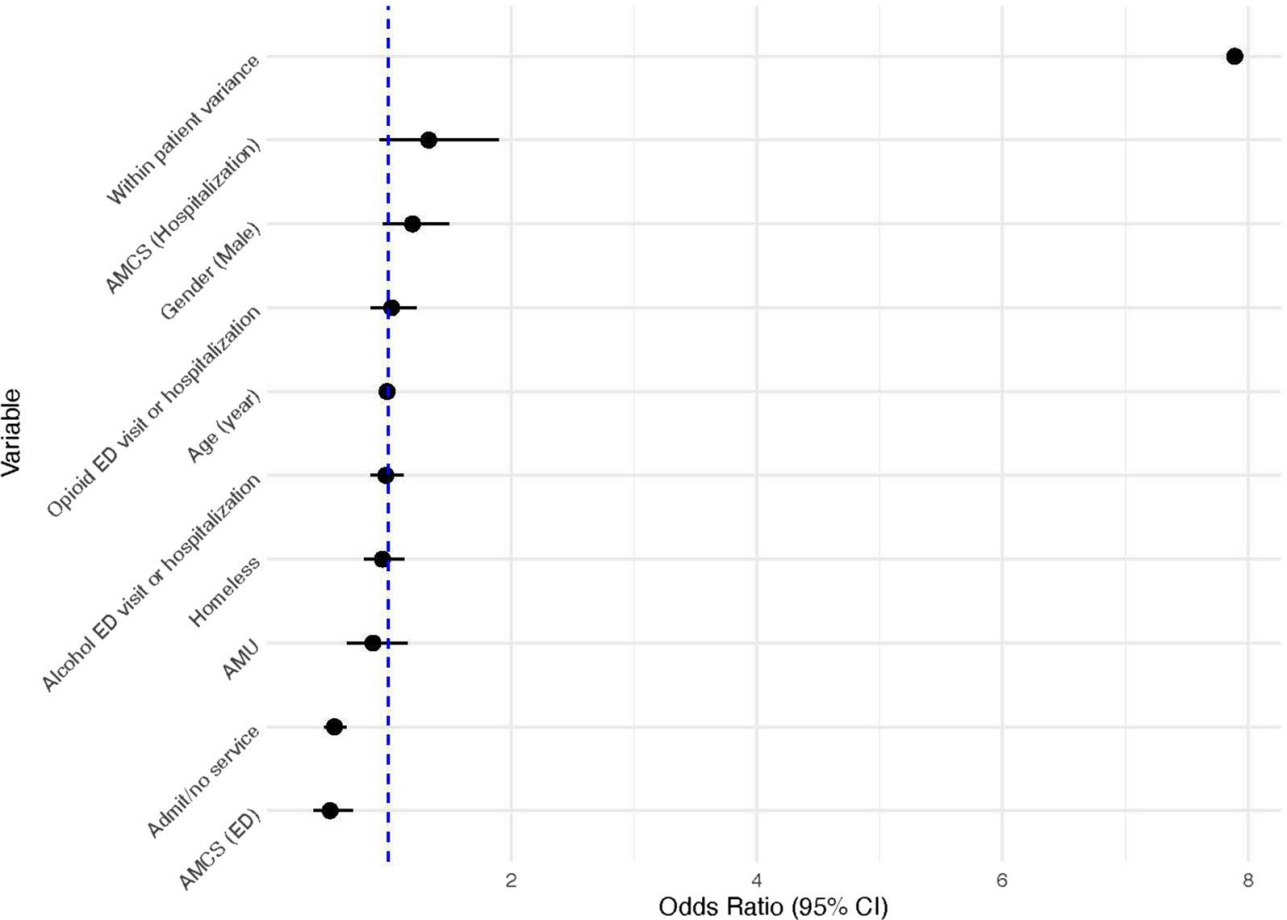
Forest plot of odds ratios for 30-day readmission

From a logistic regression model with random effects on 30-day revisits rates, the variable AMCS used in the ED showed a significant negative association with the odds of revisits (OR 0.53, 95% CI 0.39–0.71, *p* < 0.01). Similarly, the Admit/no service variable demonstrated a significant correlation with a decrease in the odds of revisits (OR 0.56, 95% CI 0.48–0.66, *p* < 0.01). Age showed an inverse relationship with revisits (OR 0.99, 95% CI 0.98–1.00, *p* = 0.01). The OR = 7.89 for the random effects in the model indicated that a past revisits was associated with a 7.89 fold increase in subsequent revisits within 30-days (Table 2, Fig. 3).

**Table 2:** Logistic regression with random effects for 30-day readmission

Variable	Odds ratio	LowerCI	UpperCI	*p* value
(Intercept)	0.04	0.03	0.07	< 0.01
AMCS (ED)	0.53	0.39	0.71	< 0.01
AMCS (hospitalization)	1.33	0.93	1.90	0.12
Admit/no service	0.56	0.48	0.66	< 0.01
AMU	0.88	0.66	1.16	0.35
Gender (Male)	1.20	0.96	1.50	0.11
Age (year)	0.99	0.98	1.00	0.01
Homeless	0.95	0.80	1.13	0.58
Opioid ED visit or hospitalization	1.03	0.86	1.23	0.76
Alcohol ED visit or hospitalization	0.98	0.86	1.12	0.79
Random effects (SD)	7.89			

*ED* emergency department, *AMCS (hospitalization)* Addiction medicine consult service during hospital admission, *AMU* Addiction medicine unit, *CI* confidence interval

**Fig. 4:**
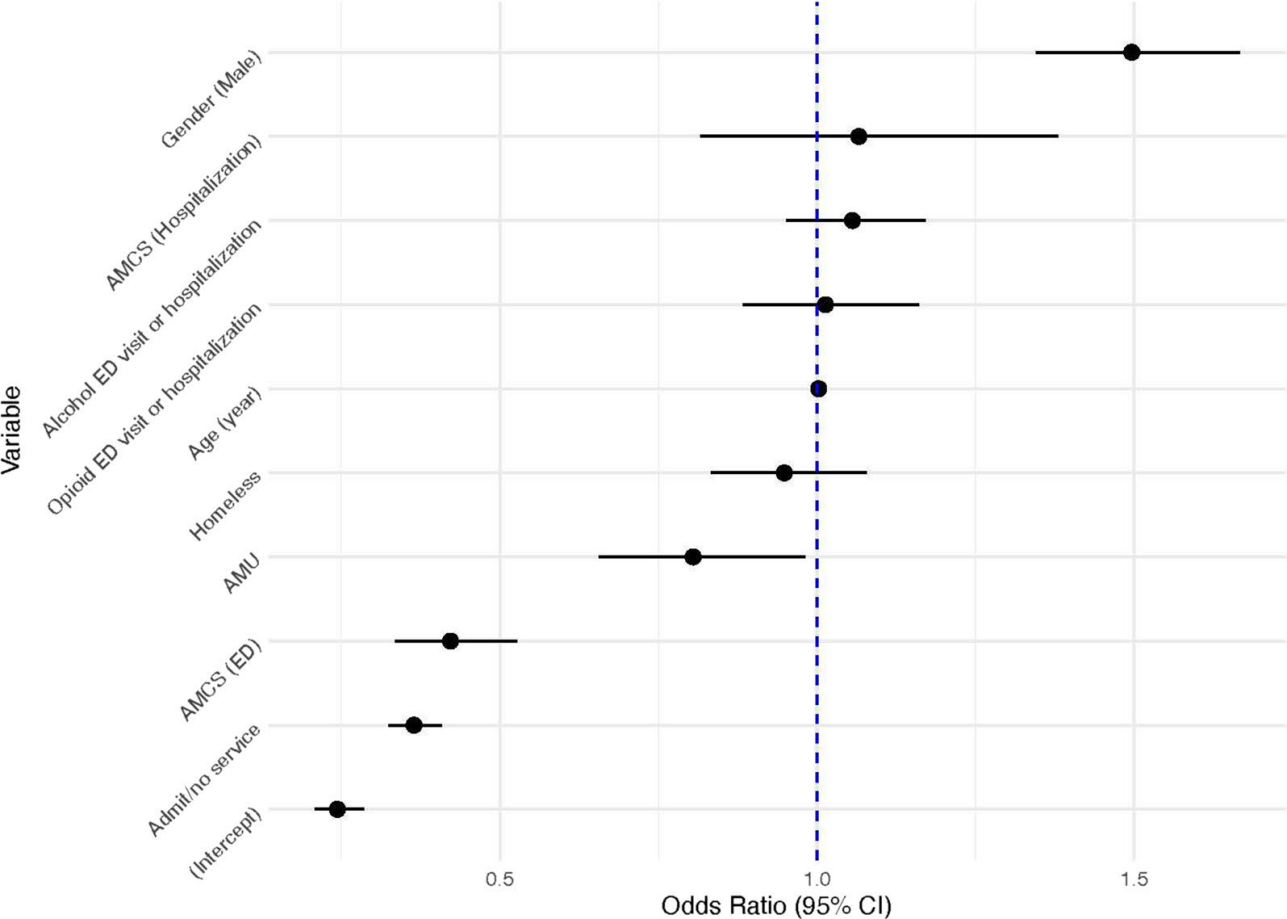
Forest plot for Odds Ratios of first readmission

The logistic regression model for first revisits showed a significant association between AMCS in ED and reduced odds of first revisits (OR 0.42, 95% CI 0.33–0.53, *p* < 0.01). The Admit/no service variable showed a statistically significant decrease in revisits odds (OR 0.36, 95% CI 0.32–0.41, *p* < 0.01). The AMU group was associated with lower revisits odds (OR 0.80, 95% CI 0.66–0.98, *p* = 0.03). Biological sex showed a statistically significant association on revisits odds, with males having an increased risk compared to females (OR 1.50, 95% CI 1.35–1.67, *p* < 0.01). (Table 3, Fig. 4).

**Table 3:** Logistic regression model for first re-admission

Variable	Odds ratio	LowerCI	UpperCI	*p* value
(Intercept)	0.24	0.21	0.29	< 0.01
AMCS (ED)	0.42	0.33	0.53	< 0.01
AMCS (hospitalization)	1.07	0.82	1.38	0.63
Admit/no service	0.36	0.32	0.41	< 0.01
AMU	0.80	0.66	0.98	0.03
Gender (male)	1.50	1.35	1.67	< 0.01
Age (year)	1.00	1.00	1.01	0.11
Homeless	0.95	0.83	1.08	0.42
Opioid ED visit or hospitalization	1.01	0.88	1.16	0.85
Alcohol ED visit or hospitalization	1.06	0.95	1.17	0.30

*ED* emergency department, *AMCS (hospitalization)* Addiction medicine consult service during hospital admission, *AMU* Addiction medicine unit, *CI* confidence interval

### Kaplan–Meier results

**Fig. 5:**
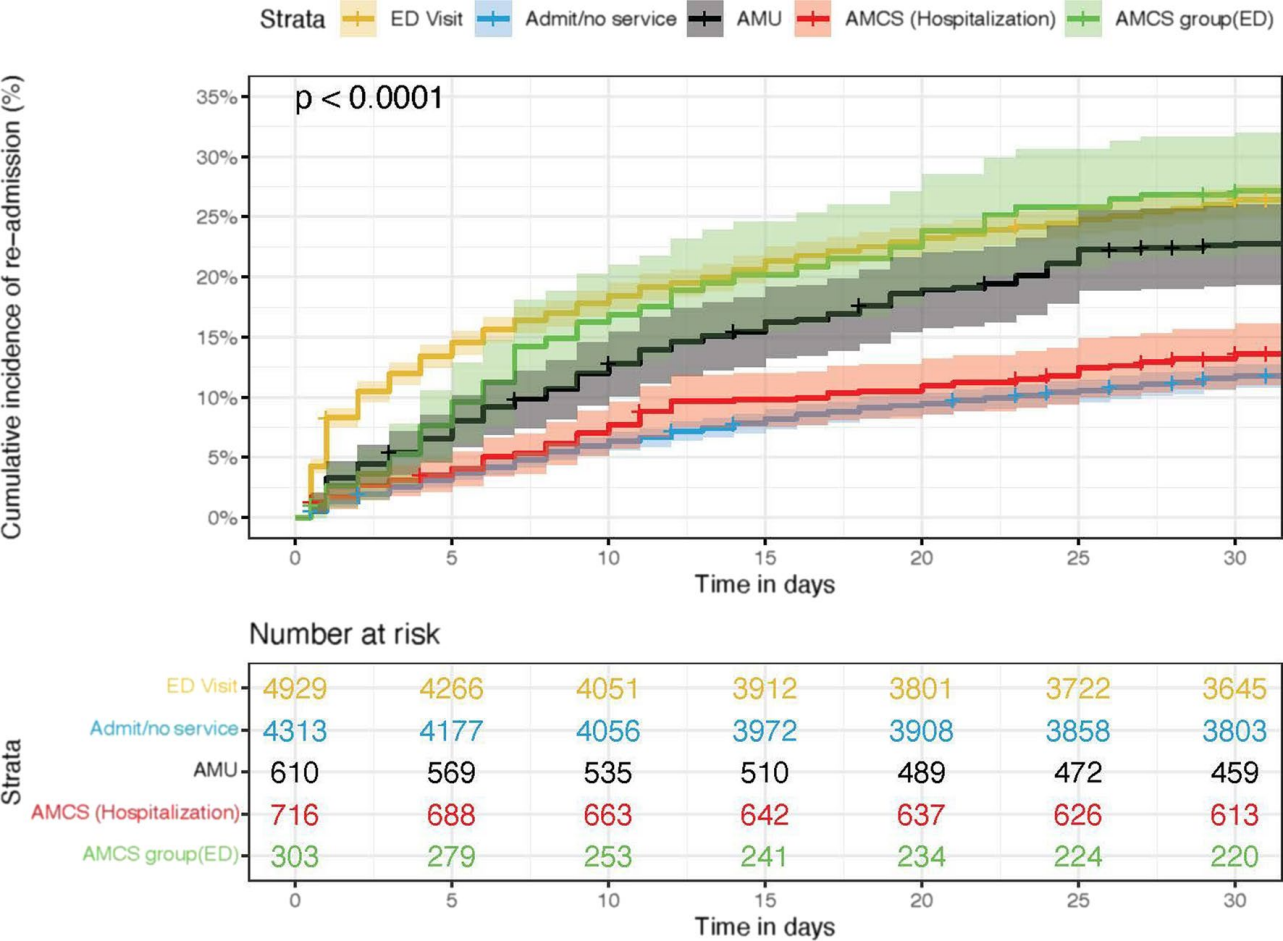
Cumulative incidence of 30-day readmission by intervention

**Fig. 6:**
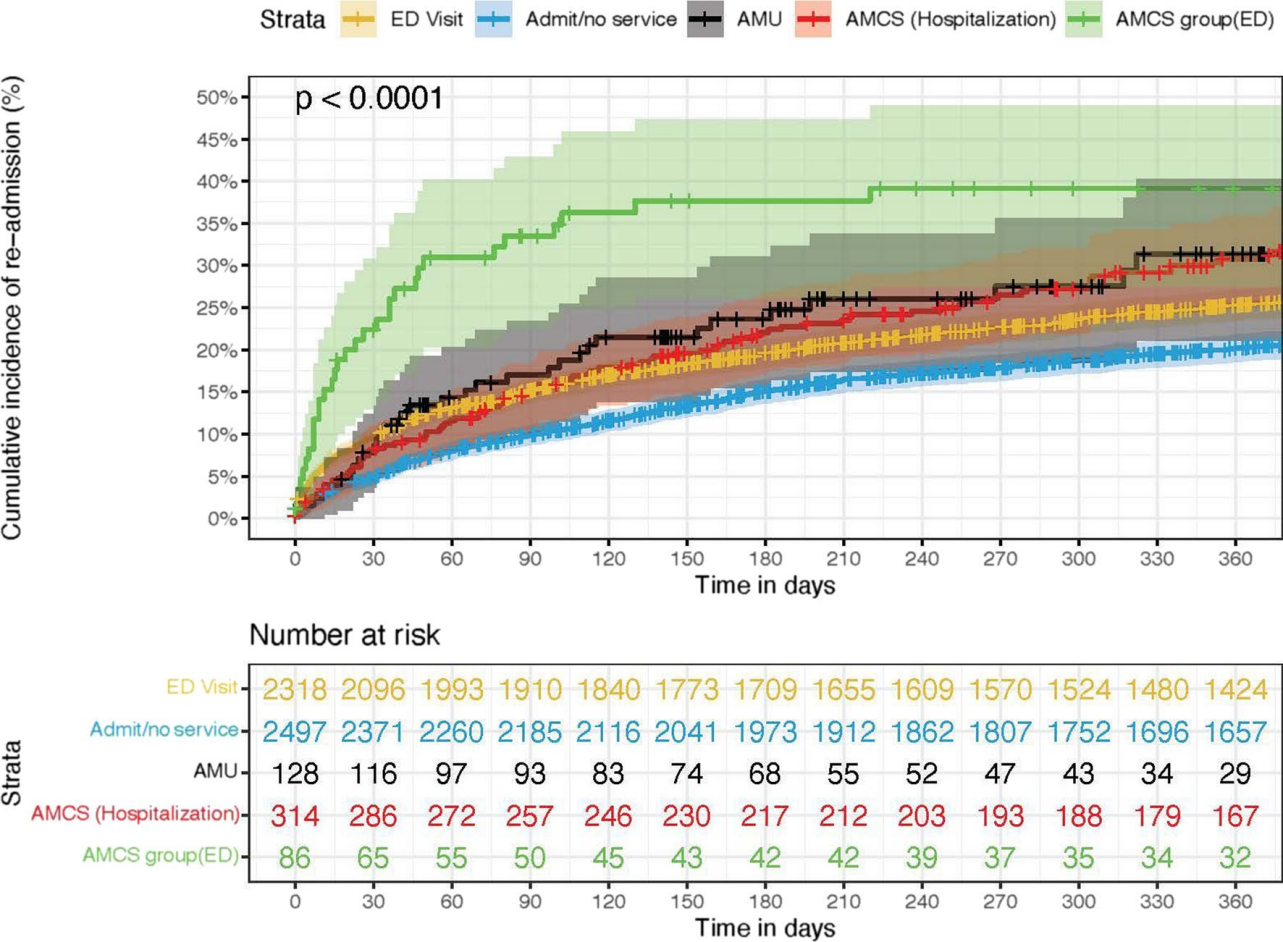
Cumulative incidence of first readmission by intervention

The time to revisit within 30-day rolling was 80.4% at day 30 (CI 0.797–0.812) with windows grouped by intervention indicating one or more statistically significant differences between the groups (Mantel-Haentzel *p* < 0.01) (Fig. 5). Cumulative incidence of first revisit was 76.3% (CI 75.2–77.5) at 1 year post index, also showed statistically significant differences between one or more groups (Mantel–Haenszel test *p* < 0.01) (Fig. 6).

### Cox proportional hazards results

#### 30-day revisits

The Cox proportional hazards model with random effects revealed an association between AMCS used in the ED and a decreased risk of 30-day revisits (HR 0.58, 95% CI − 0.76 to − 0.32, *p* < 0.01). The variable Admit/no service was also associated with a reduced risk of revisits (HR 0.60, 95% CI − 0.62 to − 0.40, *p* < 0.01). Age showed a significant relationship with revisit risk, with each year of age slightly decreasing this risk (HR 0.99, 95% CI − 0.01 to − 0.00, *p* = 0.012). Being male was associated with an increased risk of revisit (HR 1.17, 95% CI 0.00 to 0.31, *p* = 0.049). Finally, the standard deviation of the random effects within patients was 1.13, suggesting 13% variability in revisit risk at the patient level (Table 4).

**Table 4:** Cox proportional hazards model with random-effects for 30-day readmission

Variable	Hazard ratio	LowerCI	UpperCI	*p* value
AMCS (ED)	0.58	− 0.76	− 0.32	< 0.01
AMCS (hospitalization)	1.19	− 0.07	0.42	0.17
Admit/no service	0.60	− 0.62	− 0.40	< 0.01
AMU	0.83	− 0.38	0.00	0.055
Gender (male)	1.17	0.00	0.31	0.049
Age (year)	0.99	− 0.01	− 0.00	0.012
Homeless	0.98	− 0.13	0.10	0.8
Opioid ED visit or hospitalization	1.00	− 0.12	0.13	0.96
Alcohol ED visit or hospitalization	0.98	− 0.11	0.07	0.68
Random effects (within patient)	Standard deviation	Variance		
	1.13	1.28		

*ED* emergency department, *AMCS (hospitalization)* Addiction medicine consult service during hospital admission, *AMU* Addiction medicine unit, *CI* confidence interval

#### First revisit

In the Cox proportional hazards model for first re-visit, AMCS in the ED was associated with a decreased risk of first re-admission (HR 0.45, 95% CI 0.36–0.55, *p* < 0.01). The Admit/no service also showed a negative association with first revisit risk (HR 0.39, 95% CI 0.35–0.43, *p* < 0.01). Furthermore, AMU was significantly associated with reduced risk of first revisit (HR 0.80, 95% CI 0.67–0.95, *p* = 0.01). Biological sex was associated with first revisit risk, with males having a higher risk compared to females (HR 1.44, 95% CI 1.31–1.58, p < 0.01). Lastly, with each additional year of age, the risk of first revisit slightly increased (HR 1.00, 95% CI 1.00–1.01, *p* = 0.02) (Table 5).

**Table 5:** Cox proportional hazards model for first re-admission

Variable	Hazard ratio	LowerCI	UpperCI	*p* value
AMCS (ED)	0.45	0.36	0.55	< 0.01
AMCS (hospitalization)	1.02	0.82	1.28	0.86
Admit/no service	0.39	0.35	0.43	< 0.01
AMU	0.80	0.67	0.95	0.01
Gender (male)	1.44	1.31	1.58	< 0.01
Age (year)	1.00	1.00	1.01	0.02
Homeless	0.96	0.86	1.08	0.51
Alcohol ED visit or hospitalization	1.05	0.96	1.15	0.26
Opioid ED visit or hospitalization	1.01	0.90	1.14	0.87

*ED* emergency department, *AMCS (hospitalization)* Addiction medicine consult service during hospital admission, *AMU* Addiction medicine unit, *CI* confidence interval

### Interpretation

#### Summary of results

In our study, we investigated the association of two interventions and two standard care approaches for patients who had an ED visit or hospitalization related to substance use. Our research yielded several key findings: substance use interventions (AMU and AMCS) provided in the hospital setting were associated with a decreased likelihood of 30-day revisits; patients receiving AMU were more likely to have ED revisits in the long term (beyond the 30-day window); a history of revisit is a significant factor in predicting ED revisits; among the different groups studied, the ED visit group had the highest incidence of 30-day revisits, followed by the AMU, AMCS, and Admission/No service groups; the AMCS group demonstrated a significant reduction in the time to the first revisit. These findings can serve as valuable guidance for clinicians and healthcare administrators in developing treatment plans and recommending more effective, patient-centered interventions to improve outcomes for individuals with substance use-related hospital visits (Fig. 2).

#### Interpretation of findings

Our findings show heterogeneity among patients receiving addiction-related support at HSN. Patients in the AMU group were referred due complex needs, shown by higher proportions of homelessness, mental health hospital admissions, and opioid use. Our findings align with previous research indicating that patients with SUD are typically aged 35–45 years, with younger patients less likely to engage in care, as seen in the ED visits group [3].

The implementation of the AMU and the AMCS programs was associated with reduced short-term repeated health service utilization supporting the findings of previous studies [23–29]. However, our findings indicate that AMCS and AMU patients face an increased risk of ED revisits over the long term. This suggests that acute care services alone are insufficient in addressing the complex, interconnected health and social needs and the chronicity of substance use disorders [9, 30].

The group of patients admitted but did not receive addiction services showed a decreased likelihood of revisiting the hospital at both observed time points when compared to those who visited the ED but were not admitted or did not receive services. This suggests that the decision to admit patients to the hospital, rather than having them leave or be discharged directly from the ED, significantly influences patient care outcomes. Essentially, the provision of ongoing medical attention within the hospital setting appears to contribute to a reduced need for subsequent ED visits, indicating the potential benefits of inpatient care for managing patients' medical needs more effectively. Patients may be returning to the AMU and AMCS in the long term because, in comparison to standard care, the AMU and AMCS aim to create a non-judgmental environment by embracing harm reduction principles. This approach may foster a sense of trust and comfort among patients, leading them to seek care and support from the AMU even in the absence of acute medical issues. This finding is supported by research showing that social needs such as housing, social isolation, and limited social supports [31–33], along with the treatment and discharge elements that occur during a patient’s point of contact with the health care system are among the factors that account for variations in outcomes for people with SUDs seeking care [34].

### Future direction

Further investigation is required to identify causative factors contributing to the reduced risk of 30-day ED revisits. Stigma, discrimination, and healthcare provider bias may prevent people who use substances from seeking necessary care, leading to crisis-type visits outside the 30-day window. Future research should explore the intersectionality of complex health and social needs and the hospital-to-community transition to better understand high revisit rates and expand outcome collection to the full medical record (Additional file 1).

### Limitations

The study's limitations include the use of retrospective administrative data without clinical variables from patient charts, which prevents causal attributions and allows only associations between interventions and outcomes. Operationalizing covariates such as homelessness or visit reason may be susceptible to measurement error, and unobserved covariates might affect intervention-outcome associations. Variables measured at baseline are not fixed over time, and the study's generalizability may be limited to similar hospital settings. Events captured outside of HSN were not captured (i.e. death or admission to other hospitals) however, the nearest acute care facility is approximately 150 km away. Additionally, group assignment could have influenced the characteristics of the population in each intervention due to channel bias.

## Conclusion

Our study identified differences in patient populations receiving substance use support at HSN and demonstrated differences in 30-day revisits among those receiving addiction support in the hospital. We observed an unexpected finding requiring further investigation: patients in an addiction medicine unit are more likely to revisit the hospital outside the 30-day window. By understanding these distinct patient populations and the factors that contribute to revisits, healthcare providers can develop more targeted, effective interventions to support those struggling with substance use disorders.

### Supplementary Information

**Additional file 1.** Data table for Comorbidites Measured by Clinical Classifications Software Refined (CCSR).

## Data Availability

The datasets generated and/or analyzed during the current study are not publicly available because the dataset is restricted in compliance with local and national applicable health privacy laws but are available from the corresponding author on reasonable request.

## Appendix A

### Description of the addiction medicine unit (AMU)

The AMU is a 20-bed medical unit Located at Health Sciences North (HSN) in Sudbury Ontario. The unit, opened March 10th, 2023 operates based on the HSN Harm Reduction philosophy and focuses on specialized substance use care for patients at various stages of recovery. The unit addresses both the medical and psychosocial needs of patients, offering addictions-focused wrap-around care. The team comprises a specialized workforce, including physician specialists in addictions care, nursing, allied health, and peer engagement, all working together to improve outcomes. The unit is located in the hospital and it allows patients from medical and psychiatric units to be admitted for continuing medical care while receiving necessary addictions support. Admission requires a recommendation from a physician on a hospital unit and acceptance by the AMU physician. The unit provides addiction support regardless of whether a harm reduction or abstinence-based approach is followed. Additionally, it helps alleviate bed pressures for medical units and supports patients who may face challenges with outpatient therapy due to their social circumstances. The unit assists patients by ensuring they complete their medical stay and provides addictions support until discharge, establishing vital connections with community partners to ensure continued care after discharge.

### Patient flow to AMU

To ensure appropriate patient placement within the limitations of the AMU, several considerations must be taken into account. These limitations include the absence of oxygen or suction capabilities, weight restrictions due to available beds, and building code requirements that restrict the admission of wheelchair-bound patients.

From a logistical standpoint, the process begins with the sending unit identifying a patient with a substance use issue who could benefit from the services provided by the AMU. Consultation with the patient's Most Responsible Physician (MRP) takes place to seek support for the transfer. If the MRP does not support the decision, the team has the option to submit the Addiction Medicine Consult Service (AMCS) referral to provide support while the patient remains on their current unit.

If the MRP supports the transfer, an order entry is made. Subsequently, close collaboration occurs between the AMCS team, social worker, and addictions worker to assess the patient's suitability based on the admission criteria. Once the patient is deemed suitable, the patient can be transferred.

The bed flow system is then notified about the acceptance of the patient for transfer to the AMU. A handover takes place between the MRPs and a nurse-to-nurse report is provided to support seamless care transitions. Finally, the patient is transferred to the AMU, where they can receive the specialized care they require.

## Footnotes

1 Clinical Classifications Software Refined (CCSR) For ICD-10-PCS Procedures [Available from: https://hcup-us.ahrq.gov/overview.jsp

2 Goel MK, Khanna P, Kishore J. Understanding survival analysis: Kaplan–Meier estimate. Int J Ayurveda Res. 2010;1(4):274–8.
