# Supplementary material for: Association of hospital-based substance use supports on emergency department revisits: a retrospective cohort study in Sudbury, Canada from 2018 to 2022
Source: Harm Reduct J. 2024 Mar 28;21:71. doi: 10.1186/s12954-024-00985-0 (PMC10976798; doi:10.1186/s12954-024-00985-0)
Supplement: Supplementary file 1 — Additional file 1. Data table for Comorbidites Measured by Clinical Classifications Software Refined (CCSR). [file 12954_2024_985_MOESM1_ESM.pdf]

Supplement 1: Data table for Comorbidites Measured by Clinical Classifications Software Refined (CCSR)

	CCSR Clinical domain code	Exposure Group	Admission events	Total Admissions for Exposure Group	Clinical Domain Description
1	bld	AMCS (ED)	2	672	Blood, blood-forming organs, immune mechanism
2	bld	ED Visit	2	4921	Blood, blood-forming organs, immune mechanism
3	bld	Admit/no service	19	3885	Blood, blood-forming organs, immune mechanism
4	bld	AMU	2	454	Blood, blood-forming organs, immune mechanism
5	cir	AMCS (ED)	32	672	Circulatory system
6	cir	ED Visit	14	4921	Circulatory system
7	cir	AMU	10	454	Circulatory system
8	cir	Admit/no service	148	3885	Circulatory system
9	den	ED Visit	13	4921	Dental
10	den	AMCS (ED)	1	672	Dental
11	den	Admit/no service	2	3885	Dental
12	den	AMCS (Hospitalization)	1	303	Dental
13	dig	ED Visit	23	4921	Digestive system
14	dig	AMCS (ED)	58	672	Digestive system
15	dig	Admit/no service	191	3885	Digestive system
16	dig	AMU	20	454	Digestive system
17	dig	AMCS (Hospitalization)	1	303	Digestive system
18	ear	Admit/no service	3	3885	Ear, mastoid process
19	end	ED Visit	20	4921	Endocrine, nutritional and metabolic diseases
20	end	Admit/no service	105	3885	Endocrine, nutritional and metabolic diseases
21	end	AMCS (Hospitalization)	1	303	Endocrine, nutritional and metabolic diseases
22	end	AMCS (ED)	19	672	Endocrine, nutritional and metabolic diseases
23	end	AMU	14	454	Endocrine, nutritional and metabolic diseases
24	ext	Admit/no service	150	3885	External morbidity
25	ext	ED Visit	33	4921	External morbidity
26	ext	AMCS (Hospitalization)	4	303	External morbidity
27	ext	AMCS (ED)	25	672	External morbidity
28	ext	AMU	10	454	External morbidity
29	eye	ED Visit	1	4921	Eye and adnexa
30	eye	Admit/no service	1	3885	Eye and adnexa
31	fac	ED Visit	23	4921	Contact with health services
32	fac	AMU	1	454	Contact with health services
33	fac	AMCS (ED)	8	672	Contact with health services
34	fac	Admit/no service	35	3885	Contact with health services
35	gen	AMCS (ED)	16	672	Genitourinary system
36	gen	AMCS (Hospitalization)	1	303	Genitourinary system
37	gen	ED Visit	8	4921	Genitourinary system
38	gen	Admit/no service	37	3885	Genitourinary system
39	gen	AMU	5	454	Genitourinary system
40	inf	AMCS (ED)	47	672	Infectious and parasitic diseases
41	inf	AMCS (Hospitalization)	1	303	Infectious and parasitic diseases
42	inf	ED Visit	5	4921	Infectious and parasitic diseases
43	inf	Admit/no service	98	3885	Infectious and parasitic diseases
44	inf	AMU	13	454	Infectious and parasitic diseases
45	inj	AMU	18	454	Injury, poisoning
46	inj	AMCS (ED)	43	672	Injury, poisoning
47	inj	Admit/no service	185	3885	Injury, poisoning
48	inj	AMCS (Hospitalization)	3	303	Injury, poisoning
49	inj	ED Visit	123	4921	Injury, poisoning
50	mbd	AMCS (ED)	140	672	Mental, behavioral, neurodevelopmental
51	mbd	AMU	252	454	Mental, behavioral, neurodevelopmental
52	mbd	ED Visit	4280	4921	Mental, behavioral, neurodevelopmental

53	mbd	AMCS (Hospitalization)	277	303	Mental, behavioral, neurodevelopmental
54	mbd	Admit/no service	1973	3885	Mental, behavioral, neurodevelopmental
55	mus	ED Visit	16	4921	Musculoskeletal, connective tissue
56	mus	AMU	15	454	Musculoskeletal, connective tissue
57	mus	AMCS (ED)	29	672	Musculoskeletal, connective tissue
58	mus	Admit/no service	69	3885	Musculoskeletal, connective tissue
59	neo	AMU	6	454	Neoplasms
60	neo	AMCS (Hospitalization)	1	303	Neoplasms
61	neo	ED Visit	10	4921	Neoplasms
62	neo	AMCS (ED)	17	672	Neoplasms
63	neo	Admit/no service	47	3885	Neoplasms
64	nvs	ED Visit	56	4921	Nervous system
65	nvs	AMU	9	454	Nervous system
66	nvs	AMCS (ED)	39	672	Nervous system
67	nvs	Admit/no service	137	3885	Nervous system
68	prg	Admit/no service	3	3885	Pregnancy, childbirth, puerperium
69	prg	AMCS (ED)	2	672	Pregnancy, childbirth, puerperium
70	prg	ED Visit	1	4921	Pregnancy, childbirth, puerperium
71	rsp	ED Visit	10	4921	Respiratory system
72	rsp	Admit/no service	173	3885	Respiratory system
73	rsp	AMU	11	454	Respiratory system
74	rsp	AMCS (ED)	49	672	Respiratory system
75	skn	ED Visit	34	4921	Skin and subcutaneous tissue
76	skn	AMCS (ED)	81	672	Skin and subcutaneous tissue
77	skn	Admit/no service	96	3885	Skin and subcutaneous tissue
78	skn	AMCS (Hospitalization)	2	303	Skin and subcutaneous tissue
79	skn	AMU	39	454	Skin and subcutaneous tissue
80	sym	AMCS (ED)	62	672	Abnormal not elsewhere classified
81	sym	Admit/no service	357	3885	Abnormal not elsewhere classified
82	sym	AMCS (Hospitalization)	11	303	Abnormal not elsewhere classified
83	sym	AMU	23	454	Abnormal not elsewhere classified
84	sym	ED Visit	248	4921	Abnormal not elsewhere classified
